# CAESAR models for developmental toxicity

**DOI:** 10.1186/1752-153X-4-S1-S4

**Published:** 2010-07-29

**Authors:** Antonio Cassano, Alberto Manganaro, Todd Martin, Douglas Young, Nadège Piclin, Marco Pintore, Davide Bigoni, Emilio Benfenati

**Affiliations:** 1 Laboratory of Chemistry and Environmental Toxicology, Istituto di Ricerche Farmacologiche Mario Negri, Milan, Italy; 2 Sustainable Technology Division, National Risk Management Research Laboratory, Office of Research and Development, U.S. Environmental Protection Agency, Cincinnati, OH, USA; 3 BioChemics Consulting, BCX, Olivet cedex, France; 4 Davide Bigoni, Consulting Software Engineer, http://www.bigoniconsulting.com

## Abstract

**Background:**

The new REACH legislation requires assessment of a large number of chemicals in the European market for several endpoints. Developmental toxicity is one of the most difficult endpoints to assess, on account of the complexity, length and costs of experiments. Following the encouragement of QSAR (*in silico*) methods provided in the REACH itself, the CAESAR project has developed several models.

**Results:**

Two QSAR models for developmental toxicity have been developed, using different statistical/mathematical methods. Both models performed well. The first makes a classification based on a random forest algorithm, while the second is based on an adaptive fuzzy partition algorithm. The first model has been implemented and inserted into the CAESAR on-line application, which is java-based software that allows everyone to freely use the models.

**Conclusions:**

The CAESAR QSAR models have been developed with the aim to minimize false negatives in order to make them more usable for REACH. The CAESAR on-line application ensures that both industry and regulators can easily access and use the developmental toxicity model (as well as the models for the other four endpoints).

## Background

Developmental toxicity is receiving increasing attention on account of its adverse impact at the level of the species [1]. Developmental toxicity refers to any effect interfering with normal development, both before and after birth. This includes embryotoxic/foetotoxic effects such as reduced body weight, growth and developmental retardation, organ toxicity, death, abortion, structural defects (teratogenic effects), functional effects, peri- and postnatal defects, and impaired postnatal mental or physical development up to normal pubertal development. This important endpoint is more problematic to assess than other endpoints [2]. Developmental toxicity involves several aspects and a series of experimental methods can be adopted. The complexity, length, and cost of the experiments, and the late recognition of the importance of this endpoint have resulted in a low number of available studies.

The European legislation REACH requires specific assessment of developmental toxicity [3]. As part of the CAESAR project [4] we addressed developmental toxicity, in collaboration with the US Environmental Protection Agency, using quantitative structure-activity relationship (QSAR) models. QSAR models are welcome in REACH, which recognizes that new tools are needed to cope with the huge task of assessing the vast number of chemicals in the European market. Animal studies, which are at the basis of QSAR models for toxicity, cannot alone guarantee the production of all necessary data. Limits of traditional experimental methods include the costs and time they need, ethical concern about use of animals, and the relatively small number of laboratories that can do the experiments. Furthermore, current experimental methods are not universal and cannot provide suitable evaluations for certain properties. Innovation is therefore a major aim of REACH, as stated in its first article [3].

CAESAR wants to contribute to this effort to introduce new methods, which are useful in the case of developmental toxicity. Thus besides developing the model we dedicated efforts to making it available. This paper describes models for developmental toxicity obtained within CAESAR and describes the web-based platform, which contains the system for obtaining predictions automatically using the simple chemical structure.

## Results

### The CAESAR models

The CAESAR project's primary goal was to develop QSAR models and make them easily accessible and usable by anyone (regulators, manufacturers, etc). The first part of this section describes QSAR models for developmental toxicity, and the second part discusses the platform developed to make the models accessible.

Information on development toxicity is important for a general evaluation of the toxicity of chemicals. Developmental toxicity is a complex endpoint, and there is very little information. Our models, like other models for this endpoint, are based on available data. The quality of any model depends on the quality of the data used as input. The data we used was taken from Arena et al. [5], which is a collection of good quality data and was the largest we found when the project started. Moreover, it includes a heterogeneous list of chemicals, belonging to different chemical classes. The quality of this data set has been checked by the CAESAR consortium, for both the property classes and chemical structures (see experimental part). The collection starts from experimental values on animal toxicity, and human studies or case reports [6,7] on potentially teratogenic chemicals, so on this basis the possible human toxicity effect was evaluated. The original toxicity classes were further checked within the CAESAR project, as detailed in the experimental section. The modeling tool is a binary classifier, and thus the model predicts whether the chemical is toxic or not.

The validation of a QSAR model is important if a particular QSAR model is to be used for predictive purposes [8,9]. In the past most QSAR models only reported the statistical characteristics of fitting. In order to assess the models' predictive performance we developed the models using only 80% of the chemicals available as training set, and the other 20% for external validation as a test set. Regulators often prefers the use of an external test set because it can immediately show whether the model is predictive towards new chemicals [8,9]. In practice, the availability of experimental data is a major issue in many cases. In our case the chosen solution, 20% as test set was suitable considering the number of available compounds. Matthews et al. studied developmental toxicity models using 10% of the compounds to form the validation set, starting from more than 900 compounds [10]. Arena et al. did not use an external test set [5]. Besides external validation, we also used the leave-several-out method for internal validation. It is well recognized the validation of a QSAR model is important in case a particular QSAR model will be used for predictive purposes.

Within CAESAR we developed tens of predictive models for this endpoint. Here we report the results for the two models that gave the best results: a random forest (RF) model (based on 13 descriptors) and an adaptive fuzzy partition (AFP) model (based on 6 descriptors). The prediction statistics for the RF and AFP models are given in Tables 1 and 2, respectively. The definitions of the prediction statistics in Tables 1 and 2 are given in Table 3.

**Table 1:** Validation statistics from the RF model for developmental toxicity

Statistical parameters*	Fitting on the training set	Prediction on the test set
Accuracy	100%	84%
FP%	0%	41%
FN%	0%	5%
PPV	100%	85%
NPV	100%	83%
Sensitivity	100%	95%
Specificity	100%	59%

* The definition of these parameters is in Table 3.

**Table 2:** Validation statistics from the AFP model for developmental toxicity

Statistical parameters*	Fitting on the training set	Prediction on the test set
Accuracy	87%	88%
FP%	26%	18%
FN%	7%	10%
PPV	89%	92%
NPV	83%	78%
Sensitivity	93%	90%
Specifificy	74%	82%

* The definition of these parameters is in Table 3.

**Table 3:** Statistical variables of the performance of a binary classification test

Acronym	Full name	Definition/Formula
TP	True positive	Toxic compounds predicted as toxic
TN	True negative	Non toxic compounds predicted as non toxic
FP	False positive	Non toxic compounds predicted as toxic
FN	False negative	Toxic compounds predicted as non toxic
FP%	False positive rate	Ratio of non toxic compounds incorrectly classified as toxic FP/(FP + TN)
FN%	False negative rate	Ratio of toxic compounds incorrectly classified as non toxic FN/(FN + TP)
Sensitivity	Sensitivity or true positive rate	Ratio of toxic compounds correctly classified as toxic TP/(TP + FN)
Specificity	Specificity or true negative rate	Ratio of non toxic compounds correctly classified as non toxic TN/(FP + TN)
Accuracy	Accuracy or concordance	Proportion of the total number of predictions that were correct (TP + TN)/(TP + TN + FP + FN)
PPV	Positive predictive value	Ratio of the predicted toxic compounds that were correct TP/(TP + FP)
NPV	Negative predictive value	Ratio of the predicted non toxic compounds that were correct TN/(TN + FN)

The RF model shows good accuracy in the test set (84%), and in fitting (100%), with few false negatives (95% sensitivity). The AFP model shows high accuracy in the test set (88%) and in fitting (87%) with a good balance between sensitivity (90%) and specificity (82%). The results shown in Table 2 with the AFP model were obtained after removing one compound (etoposide), because the AFP model could not predict it. Indeed, the descriptor values for etoposide are outside the domain applicability of the AFP model for one descriptor.  The quality of the models also appears good with internal validation: the accuracy in cross-validation (leave-several-out) was 77% for the RF model, and 72% for the AFP. We used ten-fold cross-validation for RF, and leave-one-out for AFP. These results are better than those previously published on the same dataset [5,11]. These studies applied two different methods: CART decision tree and logistic regression. The prediction statistics for CART were as follows: accuracy 57-63%; sensitivity 58-64%; specificity 57-66%. The statistics for logistic regression were similar: accuracy 60-62%; sensitivity 60-63%; specificity 59-62%.

Other studies on developmental toxicity have modeled individual animal endpoints [10,12]. Accuracy in these cases ranged from 45 to 88% for reproductive toxicity, and sensitivity from 10 to 72% (however, for the last value accuracy was exceptionally low, only 45%). Thus, the results from the CAESAR models are good compared to those in the literature.

Thus, the CAESAR models offer an improvement over previous models based on the same set of compounds, and perform well compared with other studies on developmental toxicity. Our set of compounds includes a heterogeneous list of chemicals, from different classes. The same applies to the other models on developmental toxicity mentioned above.

However, the quality of a model itself is not sufficient for use. In most cases models presented in the literature are not used at all, because the possibility of applying them is typically limited by several obstacles. The user must proceed through a long series of steps, using programs which are often hard to find and reproduce. Typically, to apply a QSAR model the user has to calculate chemical descriptors, then apply the model algorithm to get the result [13]. However, several facts may put obstacles in this path limiting reproducibility. Some parts of the algorithm may be private and not distributed. Then, different programs to calculate chemical descriptors handle chemical structures in different ways. Thus, even apparently simple descriptors such as the number of double bonds, may provide different values when calculated with different programs or even different versions of the same software. Even worse is the situation with tri-dimensional descriptors, because in most cases they involve manual optimization or stochastic processes.

For developmental toxicity we developed tens of models. Since tri-dimensional descriptors are typically more difficult to calculate and in our case did not yield better results, the final CAESAR models included only bi-dimensional descriptors.

As part of the CAESAR project we organized a workshop (Milan, 10-11 March, 2009) [4] to discuss the results with regulatory stakeholders and industry representatives. Participants (about 130) included regulatory representatives from nearly all 25 European Union members and associated countries, and many from industry. We asked which features were most desirable for QSAR models and how to make the models more usable. This discussion led to a platform to make the CAESAR models available and some modules were added following users' recommendations.

This led to the development of a free web-based application that allows one to submit a list of compounds, execute the QSAR model of interest, and obtain results easily. So far, the developmental toxicity model developed with the RF algorithm has been implemented in the application, together with other models for the four other end-points selected for the CAESAR project.

The CAESAR application has a client-server architecture. The user accesses the application from his/her web browser, and can use the latest models available on the central server. More details are given in Additional file 1.

The model has one particularly valuable feature: the results are intrinsically-reproducible. Starting from a certain structure only one value can be obtained by all users.

### The CAESAR model platform

All CAESAR models are implemented with a similar user interface, designed to be simple and user-friendly. The user must upload the molecule structures and then all the necessary descriptors are calculated directly on the server side. The user follows three steps to execute the model.

In the first step the user loads the desired data; this can be done either by uploading a recognized molecular file (.SDF or .SMI) or by entering the structures of the compounds of interest using the simplified molecular input line entry system (SMILES) strings [14]. All the molecules are then listed in a grid, and when a desired element of the grid is highlighted, the bi-dimensional structure of the compound is shown.

In the second step, all molecules are sent to the CAESAR server for the calculation of the model. When the remote execution is terminated, the user can see the set of compounds and predictions resulting from the model in the grid. When a compound is highlighted, details about its prediction are reported, and it is possible to check the six most similar compounds found in the model database (see below for further details).

The final step lets the user save the prediction made by the model in two possible formats, plain text or portable document format - PDF. The first format reports the predictions in a tab delimited text file which can be imported into other software (such as a spreadsheet). The second format is a full report in PDF format, which reports the compound, its depiction, and the six most similar compounds in the database along with their bi-dimensional structures.

We presented the CAESAR platform in a few courses and at the CAESAR workshop. The CAESAR platform was well accepted by users from different backgrounds. All said they found it clearer and easier than other freely available tools for human toxicity. A clearer platform makes errors less likely. Furthermore, the user has no options to choose from and this makes the results with CAESAR reproducible.

### Applicability domain

An important issue for the proper interpretation of the results of a QSAR model is the applicability domain (AD), i.e. an evaluation of how suitable the model is for reliable prediction for the given compounds. Evaluation of the AD is required by REACH.

Within the CAESAR platform, the user can evaluate the AD using three approaches. The first refers to chemical descriptors or fragments related to the whole data set; the second is a similarity tool to show the six compounds of the training set most similar to the submitted chemical; the third allows an evaluation of the goodness or errors in prediction for these six compounds.

The first tool explores the whole set of compounds in the training set, and includes a remark if problems appear in relation to the overall set of descriptors or fragments. Each CAESAR model has its own method for this assessment, depending on the type of model (for example, checking the range of molecular descriptors, or application of expert-based rules, etc.). This assessment is reported with the results for the model, where each compound has a field headed "remarks". If problems are found concerning the AD, the user is warned that the prediction may not be reliable as the molecule falls outside the model's AD. In the case of the model for developmental toxicity, the platform verifies whether the descriptors of the new compounds are inside the range of the descriptors used for the training set.

Besides this tool for AD (which is based on the chemometric approach and thus on the input space, i.e.the chemical descriptors and fragments), there is a second tool in the CAESAR platform that enables the user to visualize the six molecules from the model's training set which are the most similar to a given compound. Furthermore, a similarity score tool has been implemented.

The question of formal measurement of the "similarity" between compounds is complex and has no clear solution. Several approaches have been proposed and used [15]. In the CAESAR application, we implemented an integrated index using both count descriptors and fingerprints, taking account of different aspects of chemical similarity in order to obtain a broad measurement of similarity. The index is based on four sub-indices, explained below:

• Functional Group Similarity (FGs): built as the sequence of all values of the Functional Group molecular descriptors as calculated by DRAGON [16], where each descriptor reports the number of occurrences of a particular functional group.

• Constitutional Similarity (Cs): built as the sequence of some molecular descriptors calculated by DRAGON in the Constitutional block: nH, nC, nN, nO, nP, nS, nF, nCL, nBR, nI, nB, nHM, nX; each descriptor reports the number of some kind of atom.

• Ring Similarity (Rs): built as the sequence of some molecular descriptors calculated by DRAGON in the Constitutional block: nCIC, nCIR, nR03, nR04, nR05, nR06, nR07, nR08, nR09, nR10, nR11, nR12, nBnz; each descriptor reports the count of each ring size.

• Fingerprint Similarity (FPs): built as a standard fingerprint as defined in the Daylight theory [17], with 1024 characters and deep 7. Thus it takes into account all the possible fragments of the molecule made of up to 7 elements.

These four sub-indices are used to calculate a similarity index based on the Tanimoto coefficient [18], so that four sub-indices in the interval (0,1) are obtained. The final similarity index is calculated using a utility function between these four, assigning a weight for each:

The index S is still in the interval (0,1), as required by our similarity index. The weights were chosen to ensure good distribution of the values in the interval (0,1). When the compound of interest is present in the training set 1 is obtained. In some cases 0 can be obtained for chemicals which are very different from those in the training set. If the model data set does not have a molecule similar to the one submitted, the confidence in the predicted value decreases. Since the six most similar chemicals are shown, besides the similarity score users can apply their own experience to evaluate similarity. This feature has also been added on the basis of requests from the workshop.

As a further tool, users can check the difference between the experimental and predicted values for these six similar compounds. This feature allows one to evaluate the model's validity for the specific domain in the vicinity of the chemical of interest. The user should also assess whether the possible error is towards false positive or negative, as given by the CAESAR platform. If the model under-predicts toxicity for similar compounds, it should not be used for the specific compound of interest.

The similarity tool described above and this evaluation of possible prediction errors are two very different concepts, and both checks must be satisfied. In the similarity evaluation the user can see if there are similar compounds in the training set or not, while in the second case the user can assess whether the model gives good results for them. These six compounds may not be very similar to the compound of interest, and this may raise concern about the reliability of the prediction, even if the predictions for each of the six compounds are accurate. Alternatively, there may be six very similar compounds which satisfy the first check on similarity; but of course if the predictions for these compounds are wrong, the model is not reliable in this specific case.

For the developmental toxicity model two compounds of the test set had descriptors out of the range of descriptors used for the training set. The prediction for one of them was wrong. Due to the limited number of compounds, it is impossible to draw conclusions.

Table 4 shows a summary of the similarity in the test set. A similarity score was calculated as the average value of three most similar compounds from the training and test sets for each molecule. Then the mean and standard deviation of these values were calculated for the four categories: true positives, true negatives, false positives and false negatives. Means and standard deviations are reported for all correctly predicted compounds (true positive and true negatives) and all incorrectly predicted (false positive and false negatives). The reliability of the predictions is related to the similarity scores. The average similarity for all correctly predicted compounds is significatively higher than the value for the outliers, and this was verified by a T test on the two groups (at significance of 0.95, the T test gave a p-value of 0.0001145). Thus, compounds for which similar molecules are found in the training set can be more reliably predicted. Compounds with less similar molecules in the training set could be out of the applicability domain of the model and thus subject to less reliable predictions.

**Table 4:** Similarity values of the compounds in the test set

prediction concordance	similarity score ^1^	number of compounds
True negative (TN)	0.828 +/- 0.075	10
True positive (TP)	0.829 +/- 0.089	39
False positive (FP)	0.717 +/- 0.062	7
False negative (FN)	0.716 +/- 0.087	2
Correctly predicted	0.829 +/- 0.085	49
Incorrectly predicted	0.717 +/- 0.062	9

1. Average of the averages for similarity values of the three most similar compounds (mean +/- standard deviation).

Thus these two tools and the automatic chemometric check done on the overall set of compounds (with possible remarks), constitute a battery of three independent approaches implemented in the CAESAR platform to address AD and increase the reliability of the model for use with specific chemicals.

## Discussion

According to REACH (Annex XI) [3] a QSAR model is valid if:

• The model is recognized as scientifically valid;

• The substance is included in the applicability domain of the model;

• Results are adequate for classification and labeling and for risk assessment;

• The model is adequately documented.

These aspects closely relate to what is also indicated within the OECD guidelines for validation of the QSAR models for regulatory purposes [8]. A (Q)SAR model for regulatory purposes should be associated with the following information:

1) a defined endpoint

2) an unambiguous algorithm

3) a defined domain of applicability

4) appropriate measures of goodness-of-fit, robustness and predictivity

5) a mechanistic interpretation, if possible.

The question of scientific validity present in REACH is related to the fourth OECD principle, which addresses the measurements of goodness-of-fit, robustness, and predictivity. The applicability domain requirement is explicitly mentioned in the specifications of the REACH legislation and in the fourth OECD principles. REACH also requires the model to be suitable for classification and labeling and for risk assessment. This is different from the OECD principles. REACH is very focused on the use of the QSAR model (and any other tool, not only *in silico*) for specific legislative purposes. Whereas the OECD principles are more generic in their application, and focus on the internal validity of the model more than on its use within a specific legislation. The adequate documentation required by REACH is related to the definition of the endpoint (as in the first OECD principle), and the algorithm (second OECD principle). As stated in the CAESAR website [4], the algorithm is available, which ensures maximum reproducibility.

REACH gives no recommendations about the mechanism of action (fifth OECD principle). In the specific case of developmental toxicity there is lack of knowledge of the mechanisms of toxicity. Even when modeling leads to the characterization of particular molecule fragments related to toxicity, it is not easy to build a precise mechanistic interpretation for their role in biological activity (see for example [19]).

The chemical descriptors selected in the CAESAR models (Tables 5 and 6 for the RF and AFP models, respectively) may suggest possible mechanisms. In both models, descriptors take into account topological information and electronic properties. In fact, most of the descriptors are eigenvalues from topological matrices (BEH, BEL) and spatial autocorrelations from molecular graphs (GATS, MATS, ATS). In both cases the descriptors are weighted by atom properties like electronegativity, polarizability and van der Waals volume. Explicit information about the electro-topological state seems to be important for modeling, as the remaining descriptors are E-states (SdssC, ShssNH, SsOH, Gmin) regarding particular atom groups (NH, OH) and global values [20]. Thus, it can be argued that the prediction of toxicity is related to the presence of certain groups, like secondary amines, and hydroxyl groups (related to hydrophilicity), polarizability and electronegativity (related to reactivity), and steric factors (which may play a role in reducing reactivity).

**Table 5:** The list of descriptors used in the RF model

Symbol	Definition
Icycem	Mean information on the vertex cycle matrix equality
BEHm1	Highest eigenvalue n. 1 of Burden matrix/weighted by atomic masses
BELp3	Lowest eigenvalue n. 3 of Burden matrix/weighted by atomic polarizabilities
BELv1	Highest eigenvalue n. 1 of Burden matrix/weighted by atomic van der Waals volumes
BELv8	Highest eigenvalue n. 8 of Burden matrix/weighted by atomic van der Waals volumes
GATS1p	Geary autocorrelation - lag 1/weighted by atomic polarizabilities
GATS2m	Geary autocorrelation - lag 2/weighted by atomic masses
GATS3v	Geary autocorrelation - lag 3/weighted by atomic van der Waals volumes
MATS1p	Moran autocorrelation - lag 1/weighted by atomic polarizabilities
MATS4p	Moran autocorrelation - lag 4/weighted by atomic polarizabilities
MATS4v	Moran autocorrelation - lag 4/weighted by atomic van der Waals volumes
SdssC	Sum of all (αC --) E-State values in molecule
ShssNH	Sum of all [-- NH -- ] E-State values in molecule

**Table 6:** The list of the descriptors used in the AFP model

Symbol	Definition
SsOH	Sum of all (-- OH) E-State values in molecule
Gmin	Smallest atom E-State value in molecule
BEHv1	Highest eigenvalue n. 1 of Burden matrix/weighted by atomic van der Waals volumes
BELe1	Lowest eigenvalue n. 1 of Burden matrix/weighted by atomic Sanderson electronegativities
BELp2	Lowest eigenvalue n. 1 of Burden matrix/weighted by atomic Sanderson electronegativities
ATS8m	Broto-Moreau autocorrelation of a topological structure - lag 8/weighted by atomic masses

In the CAESAR models, we paid great attention to the quality and scientific validity of the model, assessed as described above. The overall quality check also involved checking chemical structures (assessed by at least two separate laboratories) and toxicity data, which were examined by expert toxicologists in the CAESAR consortium.

The RF model, implemented on the web, was developed with the aim of minimizing false negatives (compounds that are predicted as safe when they are in fact toxic). False negative predictions have an impact on human health and the environment, and thus assessors want to avoid them. The method we propose contributes to the debate on the use of QSAR models for this particular endpoint, knowing that the number of compounds currently available is not very high, so the available data set may not fully represent certain chemicals to be assessed.

REACH does in fact require an assessment of the suitability of each specific QSAR model for the particular chemical under evaluation. As we explained above, in order to take this into account we developed special tools to assess the differences between the chemical we want to predict and the compounds present in the model. The user can see the similarity score, and see the performance of the model for the six compounds most similar to that to be predicted, and therefore has a direct feed-back and appreciation not only on the model's statistical properties (see Table 1), but also whether it is likely to be reliable for the compound under examination. There are several characteristics of the different tools for applicability domain we developed: the tools for descriptor range and similarity are a priori, based on the input space. Alternatively, the tool with associated errors relates to the output space of the model, i.e. to the toxicity property, since it reports the experimental and predicted toxicity value, and it is *a posteriori *tool, since it assesses the results obtained by the model.

The applicability domain is specifically assessed for the compound of interest, and the user has a direct evaluation of the reliability of the model for the space around the target compound. Thus the requirements of REACH are satisfied.

## Conclusions

REACH promotes innovation and requires that animal models are used only if other methods are not suitable. The use of valid QSAR methods is foreseen within REACH. Within CAESAR we developed models for developmental toxicity. Then a platform was created to make the model freely available through the web.

All models were statistically evaluated using strict criteria. Performance was superior to that of other models using the same data set. Since this data set is not very large and may limit the use of the model, we developed tools to guide the user toward a safer application. For example, the user can check the difference between the experimental and predicted value for compounds similar to the chemical of interest.

## Experimental

### Toxicity data

The developmental toxicity data employed in CAESAR project comprises 292 compounds extracted from Arena et al. [5] with their names, CAS numbers, molecular structures and toxicity classes. This developmental toxicity database was constructed by researchers in the Department of Environmental and Occupational Health at the University of Pittsburgh for computational analysis, by combining subsets of information from the Teratogen Information System (TERIS) [6] and US Food and Drug Administration (FDA) guidelines [7]. Both sources are evaluations of the existing human and animal data on potentially teratogenic chemicals, which physicians used for reference. The TERIS compilation is skewed toward a complete evaluation of the animal data whereas the FDA discussion emphasizes human studies or case reports, with reference to related animal studies. The original data set includes 293 compounds, but we had to eliminate Azatguiorube, because we found no structural information about this compound in two databases of chemical structures: Chemfinder [21], and ChemIDPlus [22]. Two CAESAR partners individually checked all chemical structures with these databases, in order to be sure that the chemical structures to be used for modeling were correct. We also corrected the name of the chemical Dotheipin into Dothiepin. Finally, we removed inorganic ions, which were present for some compounds (such as Cl^- ^and K^+^) and water molecules (present in one case). The full list of compounds is given in Additional file 2.

The developmental toxicity data set was divided into five categories, according to the FDA criteria, with the help of CAESAR Partner Liverpool John Moores University, as indicated in Table 7. Then, for developing classification models, the developmental toxicity data set was divided in two classes, i.e. non developmental toxicant (N) and developmental toxicant (D), as indicated in Table 7. Class N merges the first two FDA categories A and B, and the class D includes all compounds belonging to categories C, D and X.  We notice that the fraction of positive and negative compounds is different than in the data set use by Arena et al. [5].  As a consequence, the model is more conservative and somehow biased toward predicting positive scores.

**Table 7:** Division of the developmental toxicity data set according to the FDA Guidelines and CAESAR binary classes

FDA classes	Definition	CAESAR Binary class	Total compounds
Category A	Negative human studies		
		
Category B	Negative animal studies		
	No human studies executed	Non-developmental toxicant	91
	OR		
	Positive animal studies		
	Negative human studies		

Category C	Positive animal studies		
	No human studies executed		
	OR		
	No studies at all		
		
Category D	Positive human studies	Developmental toxicant	201
		
Category X	Animal OR human studies show abnormalities		
	AND/OR		
	Evidence of fetal risk based on human experience		

			292

Finally, the data set was split into training and test sets using rational design, by CAESAR Partner Helmholtz-Zentrum für Umweltforschung, using ChemProp [23,24]. Briefly, all compounds were sorted according to a hierarchical system of classes in relation to functional groups. Within classes, the compounds were then sorted according to halogen substitution, aromaticity, bond orders, ring contents, and number of atoms. Particular attention was paid to ordering compounds with mixed functional property groups. The test set was separated from the sorted list by keeping the relations between these compound classes in both sets as close as possible to the relations in the total set. The test set size was set to 20% of the overall data set. The selection of optimal training and test set sizes has been discussed in several papers [25,26] leading to the conclusion that while it is important to validate a QSAR model with a good external set, if the splitting leads to a training set smaller than 75-85% of the original data set, the modeling quality may be affected.

The final training and test sets include respectively 234 and 58 compounds, respectively (Table 8).

**Table 8:** Splitting of developmental toxicity compounds, as in table 7

Classes	Total compounds	Training Set	Test Set
Non-developmental toxicant	91	74	17
Developmental toxicant	201	160	41
Total number of compounds	292	234	58

All the 292 chemicals, with their experimental FDA categories and corresponding binary developmental toxicity classes, are listed in Additional file 1.

### Descriptor generation

Chemical descriptors have been calculated with the following programs: DRAGON [16], T.E.S.T. [27] and MDL [28]. Only bi-dimensional descriptors were taken into account, since tri-dimensional descriptors require optimization of the molecular structure. This choice also enabled the CAESAR web application to accept the SMILES strings as input (a very compact and easy way to handle molecule format).

### Descriptor selection

Using hundreds of descriptors can lead to false "chance" correlations between the descriptors and biological activity. To avoid this problem, a variety of methods were employed in the CAESAR project to reduce the data 'noise' (a procedure called feature selection). Different methods were used for the RF and AFP models, as below described. In both cases, selection was based on the descriptors of the compounds of the training set.

To select the chemical descriptors for the RF model we evaluated the following tools based on the software WEKA [29] or on the multilevel-self organization. The goal of multilevel-self-organization is to select the set of the most important input variables which best satisfy the final aim of modeling. This approach tightly embeds feature selection into the modeling process [30,31].

In particular, we used the following WEKA algorithms:

• Weka.attributeSelection.GreedyStepwise performs a greedy forward or backward search through the space of attribute subsets. It may start with no/all attributes or from an arbitrary point in the space. It stops when the addition/deletion of an attribute results in a decrease in evaluation statistics.

• Weka.attributeSelection.BestFirst searches the space of attribute subsets by greedy hill-climbing augmented with a backtracking facility. Setting the number of consecutive non-improving nodes, it allows control of the level of backtracking done. BestFirst can start with the empty set of attributes and search forward, or can start with the full set of attributes and search backward.

• Weka.attributeSelection.LinearForwardSelection is an extension of the BestFirst algorithm. It takes a restricted number of k attributes into account. Fixed-set selects a fixed number k of attributes, increasing k in each step when fixed-width is selected.

• Weka.attributeSelection.ClassifierSubsetEval evaluates the attribute subset on training data or a separate hold out testing set. It uses a classifier to estimate the 'merit' of a set of attributes.

• NoConstant90 is a pre-selection on the attributes. It deletes attributes with more than 90% of the same value. NoConstant90 has been used coupled with a feature selection algorithm (for example BestFirst).

In the end we chose 13 molecular descriptors for the RF model using the multileveled-self organization reported in Table 5 with a short description. They were calculated by T.E.S.T. software.

For the AFP model Hybrid Selection Algorithm (HSA) was used to select the best parameters out of the 790 molecular descriptors for classifying the chemicals by their toxicity potency. This method combines the Genetic Algorithms (GA) [32,33] concept with stepwise regression [34]. GA are inspired by Darwin's theory about evolution or population genetics. GA start with a *set of solutions *(represented by *chromosomes*) called the *population*. Solutions from one population are taken and used to form a new population. The assumption is that the new population will be better than the old one. Solutions are selected to form new solutions (*offspring*) according to their fitness - if they are more suitable, they have more chances of reproducing. GA work on the principle of repetition until some condition is satisfied (for example number of populations or improvement of the best solution).

Usually GA are applicable to problems where little information is available but they are not particularly suitable for local searches. Therefore a stepwise approach was combined with GA in order to reach local convergence [34,35], as it is quick and can find solutions in "promising" areas already identified. A specific index was applied, derived from the fuzzy clustering method, to evaluate the fitness function. This index has the following advantages. It can be calculated quite quickly and estimates the descriptor relevance by analyzing complex molecular distributions. To prevent over-fitting and poor generalization, a cross-validation procedure was included in the algorithm during the selection procedure. Thus, the original training dataset was randomly split into sub-training and validation sets so the fitness score of each chromosome was derived from the combination of the scores of the sub-training and validation sets.

The following parameters were used in processing the developmental toxicity data set: *fuzzy parameters*: weighting coefficient was set at 1.5, tolerance convergence was 0.001, number of iterations was 30 and cluster number was 6; *genetic parameters*: chromosome number was 10, chromosome size = total number of descriptors used; initial active descriptors in each chromosome = 8, crossover point number = 1, percentage of rejections = 0.1, percentage of crossover = 0.8, percentage of mutation = 0.05, number of generations = 10; *stepwise parameters*: ascending coefficient = 0.02, descending coefficient = -0.02. At the end six molecular descriptors were chosen for the AFP model; they are reported in Table 6 with a short description. They were calculated by DRAGON and MDL software.

### Model definition

Several methods have been used to develop binary classification models. They are all implemented in WEKA:

• weka-MultilayerPerceptron+Back_Propagation: a neural network that uses a backpropagation algorithm to classify instances.

• weka-Tree_RandomForest: it constructs a forest of random trees. For more information, see Breiman [36].

• weka-tree_J48: it generates a pruned or unpruned C 4.5 decision tree. For more information, see Quinlan [37].

Even though other methods gave similar values, for the best results the weka-Tree RandomForest was chosen as the algorithm for implementation of the model. Table 5 shows the list of T.E.S.T. [27] descriptors used in the RF model. The model was trained setting the following parameters: numbers of trees = 10, maximum depth of the trees = 12.

We used Adaptive Fuzzy Partition (AFP) [38] to generate models to predict the developmental toxicity class according to the binary classification from Table 7. This is a supervised classification method implementing a fuzzy partition algorithm [39] already reported in database mining issues applied to central nervous system activity [40] and validated elsewhere [41-46]. It models the relations between molecular descriptors and chemical activities by dynamically dividing the descriptor space into a set of fuzzy partitioned subspaces defined by fuzzy rules. The aim of the algorithm is to select the descriptor and the cut position, so one gets the maximal difference between the two fuzzy rule scores generated by the new subspaces. The score is determined by the weighted average of the chemical activity values in an active subspace A and its neighboring subspaces.

Let us assume the working space is an n-dimension hyperspace defined by n molecular descriptors. Each dimension i can be partitioned into L intervals I_ij_, where j represents an interval in the selected partition. Indicated by P(x_1_, ..., x_n_) a molecular vector in an n-dimensional hyperspace, a *rule *for a subspace S_k_, is derived by combining n intervals I_ij_, defined as follows [47]: if x_1 _is associated with μ_1k_(x_1_) and x_2 _is associated with μ_2k_(x_2_) ... and x_N _is associated with μ_Nk_(x_N_), then the score of the activity O for P is O_kP_, where x_i _represents the value of the i^th ^descriptor for the molecule P, μ_ik _is a trapezoidal membership function related to the descriptor i for the subspace k, and O_kP _is the biochemical activity value related to the subspace S_k_. The "and" of the fuzzy rule is represented by the *Min operator *[48], which selects the minimal value among all the μ_ik _components.

All the rules created during the fuzzy procedure are considered to establish the model between descriptor hyperspace and biochemical activities. The global score in the subspace S_k _can be represented by the following equation:(1)

where M is the number of molecular vectors in a given subspace, N is the total number of descriptors,  is the fuzzy membership function related to the descriptor i for the molecular vector P_j_, and  is the experimental activity of the compound P_j_. A classic centroid defuzzification procedure [49] is implemented to determine the chemical activity of a new test molecule. All the subspaces k are considered and the general formula to compute the score of the activity O for a generic molecule Pj is(2)

where N_subsp represents the total number of subspaces.

The AFP model was built on the training set using the following parameters: maximum number of rules for each chemical activity = 30; minimum number of compounds for a given rule = 2; maximum number of cuts for each axis = 4. The trapezoidal parameters used were: p/w_i _= 1.2 and q/w_i _= 0.9.

The AFP method gives the degrees of membership of the different classes for each compound within a 0 to 1 range. A compound is assigned to a given class if its degree of membership is superior to 0.5. The percentage of compounds correctly predicted is computed by comparing their experimental and predicted classes. Table 6 lists the descriptors used in the AFP model.

## Competing interests

The authors declare that they have no competing interests.

## Authors' contributions

AC and EB developed the Random Forest model, NP and MP developed the AFP model, TM and DY worked on the software implementation of the molecular descriptors and DB and AM worked on the CAESAR application development.

## Supplementary Material

Additional file 1 **CAESAR Application system structure**. Description of the CAESAR architecture. Click here for file

Additional file 2 **Compounds list**. List of the chemical compounds used in the models development by IRFMN and BCX. Click here for file
