# Supplementary material for: CAESAR models for developmental toxicity
Source: Chem Cent J. 2010 Jul 29;4(Suppl 1):S4. doi: 10.1186/1752-153X-4-S1-S4 (PMC2913331; doi:10.1186/1752-153X-4-S1-S4)
Supplement: Additional file 1 — CAESAR Application system structure. Description of the CAESAR architecture. [file 1752-153X-4-S1-S4-S1.docx]

**CAESAR Application system structure**

The system is based on a distributed application architecture, client-server based model, in which multiple remote clients makes service requests to a main server thought a specific communication protocol.

Both the client and server applications have been developed with Java Technology.

Java technology is an object-oriented, platform-independent, multithreaded programming environment. It is the foundation for Web and networked services, applications, platform-independent desktops, embedded devices and it is the ideal execution environment for Web Services.

On Server side, in order to collect request from clients and share information back to them, we use the Java Servlet API and Java RMI technology provided through a Java Apache-Tomcat application server. Java Servlet technology provides Web developers with a simple, consistent mechanism for extending the functionality of a Web server and accessing existing business systems.

The inter-communication between clients and server is granted by Java RMI technology over JRMP (Java Remote Method Protocol) protocol.

Remote Method Invocation (RMI) in Java gives a clean way to build distributed applications. In a complex environment like the internet, where firewalls abound, RMI can be blocked because it tries to connect to server hosts using arbitrary port numbers.

Typical firewalls on both client and server side do not allow this. In order to grant the functionalities not only on an Intranet network, but on a more wide mixed environment, we extend the RMI Socket functionalities, in order to provide a way for RMI to get through firewalls and to give it a higher performance with respect to standard HTTP tunneling, as shown in Fig. 1.


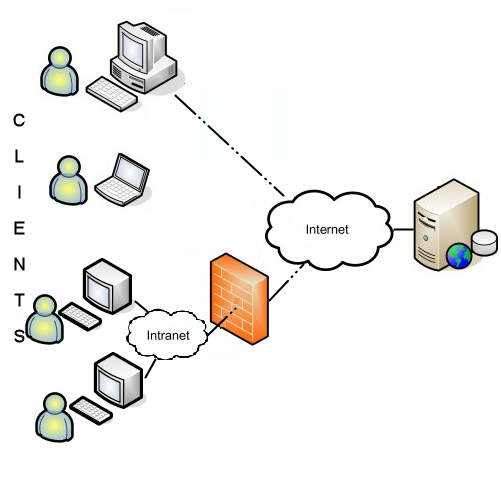


In order to be able to manage multiple concurrent requests and to be able to manage high quantity of data, the server side models are managed by a dedicated multithread server (called Model Worker Server, MWS). Each MWS is managed and launched by the application server and registered on the local RMI server registry, as shown in Fig. 2.


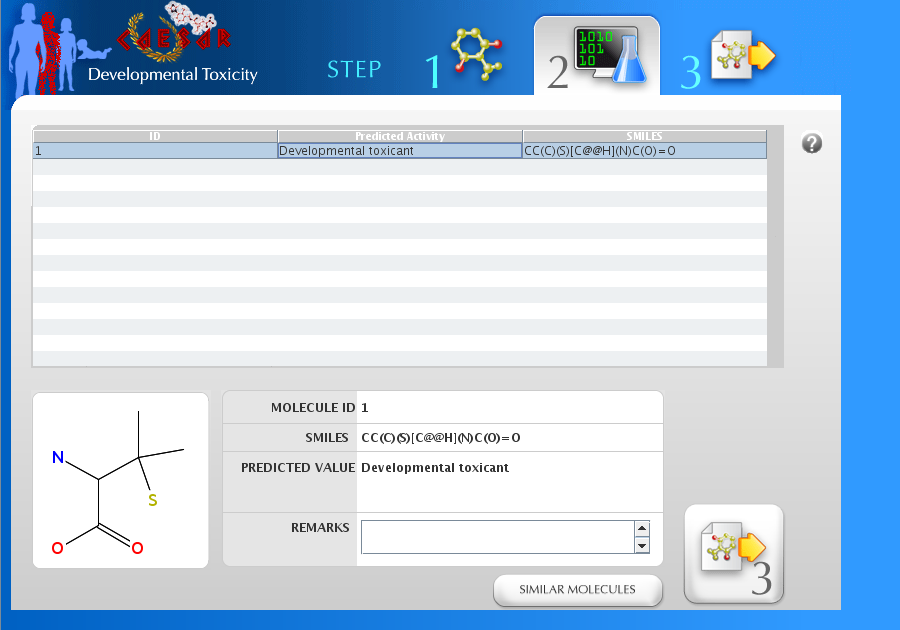


**CAESAR Application Client**

The user can access the CAESAR Application client through the official project website http://www.caesar-project.eu and download the client jnlp. The user must have an installed Java run-time environment on his/her computer, and no further installation of software is necessary.

The client automatically connects to the central server. After this connection is made, the user can choose one of the available models, one for each end-point. The client is a multitasking system, so the user is allowed to open and use more than one model.
